# Co-Occurrence of *Listeria* spp. and Spoilage Associated Microbiota During Meat Processing Due to Cross-Contamination Events

**DOI:** 10.3389/fmicb.2021.632935

**Published:** 2021-02-05

**Authors:** Benjamin Zwirzitz, Stefanie U. Wetzels, Emmanuel D. Dixon, Svenja Fleischmann, Evelyne Selberherr, Sarah Thalguter, Narciso M. Quijada, Monika Dzieciol, Martin Wagner, Beatrix Stessl

**Affiliations:** ^1^Institute of Food Safety, Food Technology and Veterinary Public Health, University of Veterinary Medicine, Vienna, Austria; ^2^Austrian Competence Center for Feed and Food Quality, Safety and Innovation FFoQSI GmbH, Tulln, Austria

**Keywords:** *Listeria monocytogenes*, meat processing, microbiome, spoilage, microbial communities

## Abstract

A large part of foodborne outbreaks related to *Listeria monocytogenes* are linked to meat and meat products. Especially, recontamination of meat products and deli-meat during slicing, packaging, and repackaging is in the focus of food authorities. In that regard, *L. monocytogenes* persistence in multi-species biofilms is one major issue, since they survive elaborate cleaning and disinfection measures. Here, we analyzed the microbial community structure throughout a meat processing facility using a combination of high-throughput full-length 16S ribosomal RNA (rRNA) gene sequencing and traditional microbiological methods. Samples were taken at different stages during meat cutting as well as from multiple sites throughout the facility environment to capture the product and the environmental associated microbiota co-occurring with *Listeria* spp. and *L. monocytogenes*. The listeria testing revealed a widely disseminated contamination (50%; 88 of 176 samples were positive for *Listeria* spp. and 13.6%; 24 of 176 samples were positive for *L. monocytogenes*). The pulsed-field gel electrophoresis (PFGE) typing evidenced 14 heterogeneous *L. monocytogenes* profiles with PCR-serogroup 1/2a, 3a as most dominant. PFGE type MA3-17 contributed to the resilient microbiota of the facility environment and was related to environmental persistence. The core in-house microbiota consisted mainly of the genera *Acinetobacter*, *Pseudomonas*, *Psychrobacter* (*Proteobacteria*), *Anaerobacillus*, *Bacillus* (*Firmicutes*), and *Chryseobacterium* (*Bacteroidota*). While the overall microbial community structure clearly differed between product and environmental samples, we were able to discern correlation patterns regarding the presence/absence of *Listeria* spp. in both sample groups. Specifically, our longitudinal analysis revealed association of *Listeria* spp. with known biofilm-producing *Pseudomonas*, *Acinetobacter*, and *Janthinobacterium* species on the meat samples. Similar patterns were also observed on the surface, indicating dispersal of microorganisms from this multispecies biofilm. Our data provided a better understanding of the built environment microbiome in the meat processing context and promoted more effective options for targeted disinfection in the analyzed facility.

## Introduction

Meat and meat products are potential vectors for foodborne pathogens, mostly *Salmonella enterica*, shiga toxin-producing *Escherichia coli*, *Campylobacter* spp., and *Listeria monocytogenes* (Fegan and Jenson, 2018; European Commission, 2020). *Listeria monocytogenes* causes listeriosis, a food-borne disease with a high case-fatality rate (15.6%) that has recently experienced a significant increase despite improvements of control measures [European Food Safety Authority and European Centre for Disease Prevention and Control (EFSA and ECDC), 2018]. A large part (5 out of 12) of foodborne outbreaks related to *L. monocytogenes* are linked to meat and meat products (EFSA Panel on Biological Hazards, 2019). Especially, recontamination of meat products and deli-meat during slicing, packaging, and repackaging is in the focus of the food control authorities (Churchill et al., 2019). The worldwide largest listeriosis outbreak in South Africa during 2017–2018 (1,060 listeriosis cases, 216 deaths) was traced back to a heat-treated meat product produced from a single meat producer (Allam et al., 2018; Kaptchouang Tchatchouang et al., 2020). Recent outbreaks have also been reported in Europe and the United States (European Centre for Disease Prevention and Control, European food safety authority, 2019; Center for Disease Control and Prevention, 2020).

The surge in listeriosis outbreaks has challenged the existing risk assessments and raised questions about the lifestyle of listeria species within the context of food processing environments (FPE). In that regard, the recurrent isolation and/or persistence of *L. monocytogenes* on food contact surfaces (FCS) and processing environments (non-FCS, NFCS) is directly related to its global spread (Nyarko et al., 2018; Harrand et al., 2020; Kaszoni-Rückerl et al., 2020). The ability of certain *L. monocytogenes* genotypes to adapt to FCS and NFCS in the FPE is of major concern. There are different factors that greatly influence the capability of *L. monocytogenes* to survive in FPE, such as their capability to adapt to low temperatures, sublethal concentrations of disinfectants, and high salt concentrations, and to grow in multi-species biofilms (Galié et al., 2018; Rodríguez-López et al., 2018; Alvarez-Ordóñez et al., 2019; Rodríguez-Melcón et al., 2019).

Insights on the behavior of *L. monocytogenes* in mono-or multi-species biofilms are gained from static or continuous flow models, but the presence of *L. monocytogenes* in FPE biofilms and the potential role of the co-existing microorganisms have not been fully explored (da Silva Fernandes et al., 2015; Dzieciol et al., 2016; Ripolles-Avila et al., 2019; Mendez et al., 2020; Rodríguez-López et al., 2020; Wagner et al., 2020). *Pseudomonas*, *Serratia*, *Brochothrix*, *Psychrobacter*, *Acinetobacter*, *Staphylococcus*, lactic acid bacteria, and aerobic and anaerobic spore-formers contribute to the resilient microbiota in meat processing plants (Stellato et al., 2016; Fagerlund et al., 2017). Understanding how the individual members of microbial communities influence each other is key in developing foodborne pathogen mitigation strategies (Van Reckem et al., 2020). It is therefore essential to investigate the microbial diversity in food processing plants and the potential relationships between the microbial community compositions with the occurrence of *L. monocytogenes* (Tan et al., 2019). The development of high-throughput sequencing technologies made it possible to study microbial populations in their natural environment, enabling researchers to gain snapshots of the world of microorganisms from broader and deeper perspectives (Cao et al., 2017; Van Reckem et al., 2020).

Several studies have reported the importance of the co-existing microbiota for *L. monocytogenes* establishment and survival. For example, Spor et al. (2020) reported that the composition of the soil microbiota is critical for the prevention of *L. monocytogenes* establishment. *Streptomyces* and *Brevibacillus* were described to produce secondary metabolites or bacteriocins with inhibitory effects on *L. monocytogenes*. Resident microorganisms of FPE (e.g., *Lactobacillus plantarum*, *Pseudomonas fluorescens*, *Pseudomonas putida*, *Enterobacteriacae*, or sulfide-producing bacteria) were shown to either protect or inhibit *L. monocytogenes* after sanitation (Rodríguez-López et al., 2018). This ambiguous behavior led us to investigate the characteristics of listeria presence in the context of whole microbial communities. We hypothesize that specific microbial community members are highly abundant in the presence of listeria in a meat cutting plant and act together to contribute to its persistence.

Therefore, we characterized the microbiological status, including the *Listeria* spp. and *L. monocytogenes* occurrence, by applying microbiological (ISO reference methods) and molecular biological methods [16S ribosomal RNA (rRNA) gene sequencing, *L. monocytogenes* serogroup PCR] in a meat cutting plant. Long read high-throughput sequencing technologies were applied to capture the complexity of microbial populations associated with the presence or absence of listeria. The study aims to provide insights into the presence of listeria and the associated microbiota during meat processing and indicates potential dynamic interactions between them. Thus, our results foster the development of novel concepts for control measures to reduce listeria presence on meat cuts delivered for the production of ready-to-eat meat products.

## Materials and Methods

### Facility Structure and Sampling

Samples for this study were taken at a large-scale meat cutting facility with a cutting capacity of 700 pork halves per hour in Austria in August 2017. Throughout the day, 10 carcass halves from three different producers (named A, B, and C) were monitored. Each half was marked upon arrival at the meat cutting plant during offloading at the cooling chamber and then subjected to primal cutting one by one, so that the same pieces could be followed and re-sampled throughout the entire processing chain (Supplementary Figure S1). At primal cutting each carcass half was cut into shoulder, leg, belly, and loin portions, which were then passed to a separate processing line. In this study, the leg and loin portions were focus of longitudinal sampling. The legs were pre-cut and held in the cooling chamber before re-joining the processing line. After cutting, the finished cuts were put in clean transport boxes, transported to the second floor, and vacuum packaged.

Carcass, meat, and environmental samples were taken with sterile polyurethane sponges using 100 cm^2^ templates (Item number SR-10NB-HDPUR-G, World Bioproducts, Woodinville, United States). First, a sterile template was placed on the sampling area then, wearing fresh sterile gloves, the hydrated sponge was taken out of the sample bag, swabbed for 10 s horizontally, then flipped and swabbed again for 10 s in vertical direction. Subsequently, the sponge was placed back into the sample bag, which was sealed and stored in the cooling chamber of the facility (4°C) until sampling was finished. Immediately after sampling, all samples were transported to the laboratory on ice to process them on the same day. Sponges were manually squeezed inside the bag to release fluid (~8 ml), which was pipetted into two separate 15 ml falcon tubes in equal proportions. One of the tubes was directly used for microbiological investigations and the other one was stored at −20°C for DNA-extraction on a later date. In total, 176 sponge samples were taken from different processing positions (product – P, *n* = 88; food contact surfaces – FCS, *n* = 48; personnel – PE, *n* = 32; non-FCS – NFCS, *n* = 8). Microbiome analysis were performed by using 16S rRNA gene amplicon sequencing on a total of 113 samples composed of 47 meat samples (carcass associated, *n* = 10; leg associated, *n* = 19; loin associated, *n* = 18) and 66 environmental associated samples (FCS, *n* = 32; PE *n* = 32; NFCS, *n* = 2). These 113 samples showed higher microbial loads according to the microbiological investigation and were therefore selected for the microbiome analysis.

### Microbiological Investigation and DNA Extraction for Isolate Confirmation

The enumeration of aerobic mesophilic counts (AMC; ISO 4833-2:2013) and hygiene indicator bacteria (Enterobacteriaceae – EB; ISO 21528-2:2017 and Pseudomonadaceae – PS) was performed after preparing a 10-fold serial dilution in buffered peptone water, (Thermo Fisher Scientific Inc., Oxoid Ltd., Basingstoke, United Kingdom) up to dilution −10^8^. The dilutions were plated in duplicates on Plate Count agar (PCA, Thermo Fisher Scientific Inc., Oxoid Ltd.), Violet Red Bile Glucose agar (VRBG, Thermo Fisher Scientific Inc., Oxoid Ltd.), and Glutamate Starch Phenol Red agar (GSP, Merck KGaA; Darmstadt, Germany) by surface plating technique. GSP and VRBG agar were incubated at 25 and 37°C for 24–48 h. PCA was incubated at 30°C for a maximum of 72 h. To determine the AMC/EB and PS counts/cm^2^, microbial colonies between 10 and 300 colony forming units (CFU) were included in the calculation. Presumptive EB and PS isolates (*n* = 5 each) were confirmed by Oxidase reaction, biochemical profiling up to genus level for PS, up to species level for EB (API 20E, API Rapid ID32E; bioMérieux Marcy-l’Étoile, France).

Listeria species detection and isolation was performed according to ISO 11290-1, modified by prolonged incubation of half Fraser enrichment to 48 h in order to increase the detection limit of the technique (ISO 11290-1, 2017). Swabs were enriched in 50 ml Half-Fraser broth (HFB; Merck KGaA) for 48 h at 30°C. After incubation, 0.1 ml HFB was transferred to 10 ml Fraser broth (FFB; Merck KGaA) and incubated for 48 h at 37°C. HFB and FFB were streaked on listeria agar acc. to Ottaviani and Agosti (ALOA; Merck KGaA). Up to five *L. monocytogenes* typical colonies and/or two *Listeria* spp., typical colonies without *L. monocytogenes* specific phosphatidylinositol-specific phospholipase C reaction were subcultivated on Tryptone Soy agar supplemented with 0.6% (wt/vol) yeast extract (TSAY; Oxoid Ltd.) for isolate based PCR confirmation and cryo-conservation. The DNA extraction for bacterial isolates was performed according a protocol published by Walsh et al. (2013). Briefly, 1–2 bacterial colonies were suspended in 100 μl 0.01 M Tris HCl Buffer (Sigma Aldrich Corp., St. Louis, MO, United States) and centrifuged for 5 min at 8,000 rpm. Subsequently, 400 μl Chelex® 100-Resin (BioRad, Hercules, CA, United States) was added to the bacterial suspension, heated for 10 min at 100°C on a thermoblock, and centrifuged at 14,000 × rpm for 5 s. The DNA supernatant was transferred to sterile tubes and stored at −20°C.

### *Listeria* spp. PCR-Based Confirmation, and *Listeria monocytogenes* Serogroup PCR

*Listeria* spp. was differentiated by a PCR-multiplex approach targeting the invasion-associated protein (iap) gene. The PCR resulted in *L. monocytogenes*, *Listeria innocua*, the *Listeria seeligeri-Listeria welshimeri-Listeria ivanovii* group, or *Listeria grayi*, respectively (Bubert et al., 1999). The gel electrophoresis of PCR-amplicons was performed in a 1.5% agarose gel containing 0.5× Tris-Borate-EDTA (TBE) buffer and 3.5 μl peqGREEN DNA gel stain (VWR International, Radnor, United States), at 120 V for 25 min. The DNA standard Thermo Scientific™ GeneRuler™ 100 bp (Thermo Fisher Scientific Inc., Waltham, United States) was applied for fragment length comparison. The *L. monocytogenes* isolates were further confirmed by serogroup PCR according to Doumith et al. (2004) and Leclercq et al. (2011) by targeting the marker genes *lmo*0737, *lmo*1118, *ORF*2819, and *ORF*2110 and the *Listeria* spp. specific *prs* gene (Doumith et al., 2004; Leclercq et al., 2011).

### *Listeria monocytogenes* Pulsed-Field Gel Electrophoresis

The genetic correlations among *L. monocytogenes* isolates were analyzed by pulsed-field gel electrophoresis (PFGE) according to CDC PulseNet standardized protocol (https://www.cdc.gov/pulsenet/PDF/listeria-pfge-protocol-508c.pdf; accessed on: 14-10-2020). The DNA fragments were digested with restriction enzyme *Asc*I (50 U, 37°C, 4 h; Thermo Fisher Scientific Inc., Waltman, MA, United States) and electrophoretically separated using CHEF-DR III system (Bio-Rad Laboratories Inc., Hercules, CA, United States). The universal standard *Salmonella* ser. Braenderup H9812 was digested with 50 U *Xba*I (Thermo Fisher Scientific Inc.) at 37°C for 4 h. PFGE results were analyzed using BioNumerics 6.6 software (Applied Maths, Sint-Martens-Latem, Belgium), and similarity between the *Asc*I macrorestriction profiles was determined with Dice coefficient (position tolerance 1.5%). Clustering and construction of dendrograms were performed by using the unweighted pair-group method with arithmetic averages (UPGMA). PFGE types were considered identical when the patterns were indistinguishable. The Simpson’s Index of diversity was calculated with the online tool of Comparing Partitions (http://www.comparingpartitions.info/; accessed on: 14-10-2020).

### DNA-Extraction and 16S rRNA Gene Sequencing

Samples selected for microbiome analysis were centrifuged at 3,220 × rcf for 20 min, and the pellet was resuspended in 400 μl of 1 × phosphate buffered saline (PBS) before DNA-extraction, in order to increase microbial cell density and neutralize inhibiting sanitizers. The DNA was then extracted from 200 μl with the QIAamp® DNA Stool Mini Kit (Qiagen GmbH, Hilden, Germany) according to manufacturer instructions. The elution step of the protocol was modified; performed two times with 50 μl DEPC treated water instead of one time with 200 μl AE buffer. Negative controls (DEPC treated sterile water), one for each used kit, were extracted together with the rest of the samples. The DNA concentration of the samples was measured with the Qubit dsDNA HS Assay Kit and Qubit 2.0 Fluorometer (Invitrogen, Thermo Fisher Scientific, Oregon, United States).

The near-full-length 16S rRNA gene amplicon libraries were prepared and sequenced according to the official Pacbio guidelines^1^ at the Vienna Biocenter Core Facilities NGS Unit.^2^ Amplicons were prepared from 113 samples (plus three negative controls) using bacteria specific primers 27F (5'-AGRGTTYGATYMTGGCTCAG-3') and 1492R (5'-RGYTACCTTGTTACGACTT-3'). In a second round of amplification, barcodes were added with Pacbio Barcoded Universal primers, so that the amplicons could be multiplexed on three SMRT cells. Sequencing was carried out on a Pacbio Sequel machine with 2.1 chemistry.

### Sequence Processing, Analysis, and Statistics

On average, each SMRT cell yielded 5,541,444 subreads which were used for circular consensus sequence (ccs) generation. The ccs command from the bioconda package pbccs v3.1^3^ was run with the minimum predicted accuracy set to 0.99 and the minimum number of passes set to 3, resulting in a mean of 246,017 ccs reads per SMRT cell. The following sequence processing pipeline was then run on files from each SMRT cell individually. Sequences were demultiplexed and then further processed through the long-read *DADA2* pipeline v1.14 in the R environment v3.6.2 (R Core Team, 2019) which measures the full-length 16S rRNA gene with single-nucleotide resolution and a near-zero error rate (Callahan et al., 2016, 2019). First, primers were trimmed with “removePrimers” and low-quality sequences were filtered using “filterAndTrim” with a maximum number of expected errors of 2. Then, the remaining reads were dereplicated and used to learn the error rates. Amplicon sequence variant (ASV) tables were generated after applying the sample inference algorithm to the dereplicated data. In the next step, individual ASV tables were merged and chimeras were removed. Finally, ASVs were classified to the SILVA rRNA database version SSU 138 (Quast et al., 2013).

All statistical analysis of the microbiome sequencing data was performed in R v3.6.2. Initial data exploration, filtering, and basic microbial community analysis were conducted using the R package *phyloseq* v1.22.3 (McMurdie and Holmes, 2013). Samples with low sequencing depth (less than 200 reads per sample) and ASVs represented by less than five reads were excluded from the analysis. Furthermore, potential contaminant ASVs were identified and removed by using the *decontam* v1.1.1 package, using a presence-based contaminant identification with a threshold of 0.5, which identifies all sequences that are more prevalent in negative controls than in positive samples as potential contaminants (Davis et al., 2018). Alpha diversity indices for the investigation of microbial communities’ richness (Chao1 index; Chao, 1984), diversity (Shannon index; Shannon, 1948), and evenness (Simpson index; Simpson, 1949) were calculated and compared with *vegan* v2.5-6 (Oksanen et al., 2011) with pairwise comparisons using Wilcoxon rank sum tests and Benjamini-Hochberg adjustment for *p*-values. For beta diversity, the “adonis” function was applied to calculate a permutational analysis of variance (PERMANOVA) with 5,000 permutations. The ASV counts of each sample were set as the dependent variable and the factors “Type,” “Position,” “Listeria,” and “Carcass” were used as independent variables. The dissimilarity in community composition was visualized in *ampvis2* v2.6.6 by means of a non-metric MultiDimensional Scaling Analysis (NMDS) based on Bray-Curtis dissimilarities as a distance measure (Andersen et al., 2018). The relative abundances of individual taxa were calculated and illustrated as barplots (Phyla) or heatmaps (Top 50 ASVs) in *phyloseq* and *ampvis2*, respectively. Shared ASVs between meat samples entering and leaving the facility were computed with the function “amp_venn” with an abundance cutoff of 0.01 and a frequency cutoff of 10 in *ampvis2*. The cutoff values were chosen to exclude potential sequencing artifacts and include ASVs that were observed in the majority (80%) of samples from one sampling position.

Correlations between individual taxa and the presence of listeria were calculated by applying spearman rank correlations in the function “associate” from the package *microbiome* v1.11.2 (Lahti and Shetty, 2017). Furthermore, taxa with significant differential abundance between samples in which *Listeria* spp. were present or absent were identified and visualized with the R package *DESeq2* v1.26 (Love et al., 2014). Only statistically significant differences (*p* < 0.05) were plotted. *Chryseobacterium* and *Psychrobacter* related ASVs that were identified in the *DESeq2* analysis were aligned and classified using the Silva Incremental Aligner v1.2.11 including 25 neighbors per query sequence (Pruesse et al., 2012). The phylogenetic trees were calculated using the maximum likelihood method implemented in MEGA X with the number of bootstrap replicates criteria set to 500 (Kumar et al., 2018). The Kimura 2-parameter model of sequence evolution was used for tree reconstruction (Kimura, 1980).

## Results

### Aerobic Mesophilic and Pseudomonadaceae Counts Increased in the Course of Processing

The AMC, *Pseudomonas* (PS), and *Enterobacteriaceae* (EB) counts on meat samples were on average 4.96, 2.32, and 2.41 log CFU/cm^2^ at the time of their delivery to the cooling chamber at the meat cutting plant (AMC range 4.48–5.40 log CFU/cm^2^; PS range < 1.0–4.85 log CFU/cm^2^; EB range < 1.0–4.48 log CFU/cm^2^; Supplementary Figure S2).

Aerobic mesophilic counts increased by 2.38 log during cutting of leg samples (7.34 mean log CFU/cm^2^) to critical limits (as stated in DGHM, 2014; 6.7 log CFU/cm^2^) and by 0.22 log (5.18 mean log CFU/cm^2^) on loin samples. Environmental samples harbored 5.17 log CFU AMC/cm^2^ on average. *Pseudomonas* levels showed a similar distribution pattern across the facility, but had a higher variation among them, i.e., lower numbers at the beginning of the processing line (Cooling chamber: 2.32 log CFU/cm^2^), and higher numbers at the end (leg after packaging: 4.78 mean log CFU/cm^2^ and loin after packaging: 4.21 mean log CFU/cm^2^). On the contrary, *Enterobacteriaceae* had relatively high numbers in the cooling chamber (2.41 mean log CFU/cm^2^) but were not detected during meat cutting and packaging. On environmental samples, *Enterobacteriaceae* was detected in samples collected from the wall in the cooling chamber, the conveyor belt at prime dissection and on hands and shoes of employees at the locks. In 10.2% (9/88) and 13.6% (12/88) of meat samples, the *Enterobacteriaceae* and *Pseudomonas* spp. warning and guide values of the DGHM recommendation (5 and 6 log CFU/g) were exceeded.

### *Listeria* spp. and *Listeria monocytogenes* Confirmation and Subtyping

All samples (*n* = 176) were tested for the presence of listeria species according to ISO 11290-1. Half of the samples were confirmed by PCR method as *Listeria* spp. (*n* = 88). About 82 (46.6%) and 24 (13.6%) of the samples were contaminated by apathogenic *Listeria* spp. and *L. monocytogenes*, respectively. Thereof, 18 samples (10.22%) contained both apathogenic listeria species and *L. monocytogenes*. The minority of samples was contaminated by exclusively *L. monocytogenes* (*n* = 6; 3.4%).

**Figure 1:**
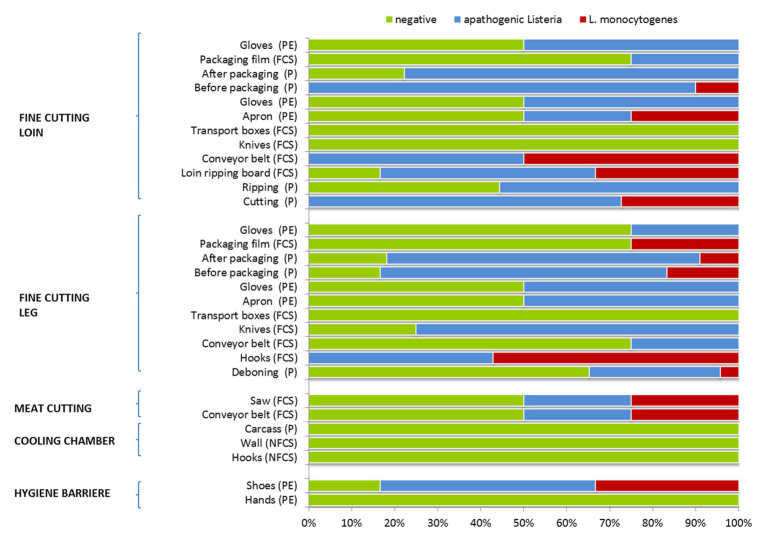
Occurrence of apathogenic listeria and *Listeria monocytogenes* during meat cutting. Number of samples positive or negative for *Listeria* spp. for each sampling position. Sample categories are abbreviated: “product” (P), “food contact surface” (FCS), “non-FCS” (NFCS), and “personnel” (PE).

Interestingly, listeria was absent from carcass samples (producers A–C), hooks and wall on delivery to the cooling chamber, but was present due to cross-contamination events during meat cutting and packaging (Figure 1). Samples collected from the surface of equipment, machines or the processing environment showed a heterogeneous distribution of listeria, indicating a wide dissemination through the facility. Initial personnel associated samples (PE) tested positive for *Listeria* spp. and *L. monocytogenes* were shoes after passing the hygiene barrier. The saw and conveyor belt at meat cutting was the next contaminated environmental sampling site (FCS). At the station for fine cutting of the leg hook were heavily contaminated with *Listeria* spp. and *L. monocytogenes* (no negative sample). The leg was cross-contaminated from the environment at deboning, before and after packaging. *Listeria* spp. was present on conveyor belts, knives, and personnel associated samples (gloves, apron). Following sampling sites were tested *Listeria* spp. and *L. monocytogenes* positive at the station for fine cutting of the loin: the meat cuts, the ripping board (FCS), conveyors (FCS), aprons (PE), and the meat before packaging. Apathogenic *Listeria* spp. was detected on products after ripping, packaging, packaging film (FCS), and gloves (PE).

**Figure 2:**
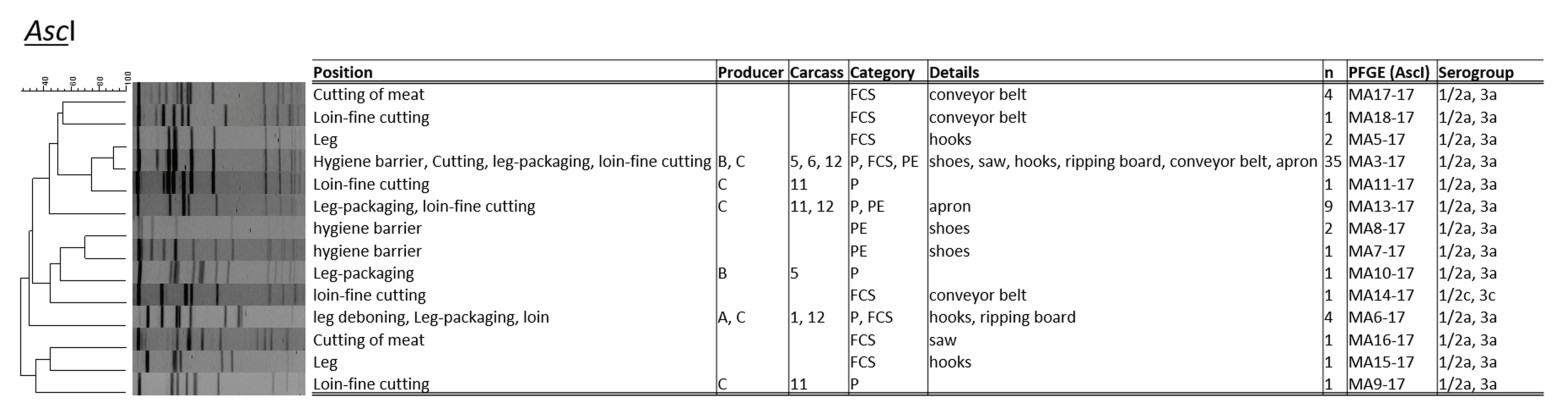
Pulsed-field gel electrophoresis (PFGE) cluster analysis with restriction enzyme *Asc*I for *L. monocytogenes* (*n* = 58) isolated during meat cutting.

The PCR serotyping of 64 *L. monocytogenes* isolates resulted to a majority in PCR serogroup 1/2a, 3a, except for one isolate (1/2c, 3c; both genetic lineage II; Figure 2). The PFGE typing applying *Asc*I revealed 14 distinct *L. monocytogenes* profiles with a Simpson’s Index of Diversity of 0.679 (CI 95%, 0.559–0.798). About 54.2% (*n* = 13/24) of *L. monocytogenes* positive samples harbored up to two or three different PFGE types in parallel. The most abundant PFGE profile was MA3-17 (*n* = 35 isolates; 54.7%), followed by MA13-17 (*n* = 9; 14.1%), and MA6-17 (*n* = 5; 7.8%). The UPGMA cluster analysis applying the dice coefficient indicated a similarity of 90% for *L. monocytogenes* MA3-17 and MA5-17. *Listeria monocytogenes* PFGE profile MA3-17 was present on shoes after passing the hygiene barrier, on the saw during cutting (FCS), hooks at the fine cutting of legs (FCS), at the packaging site of legs, and at the fine cutting of loin (FCS-conveyor belt, PE-aprons), indicating cross-contamination from the environment and personnel as vectors. MA3-17 may already contribute to more than one cross-contamination event and can be related to environmental persistence. The PFGE profile MA13-17 only appeared during the fine cutting of the loin [including a personnel vector (apron)] and at the packaging site of the leg. Profile MA6-17 appeared initially at the hooks of the leg, at the leg after deboning and at packaging. Additionally, PFGE profile MA6-17 was found at the ripping board of the loin. The hooks of the leg indicated inefficient sanitation due to the presence of up to four different PFGE-profiles (MA3-17, MA5-17, MA6-17, and MA15-17). The shoe sanitation at the hygiene barrier did not properly remove PFGE-profiles MA3-17, MA7-17, and MA817. Saw and conveyor belts also contained heterogeneous *L. monocytogenes* PFGE-types.

### The Microbial Community Structure on Meat Changes During Processing

**Figure 3:**
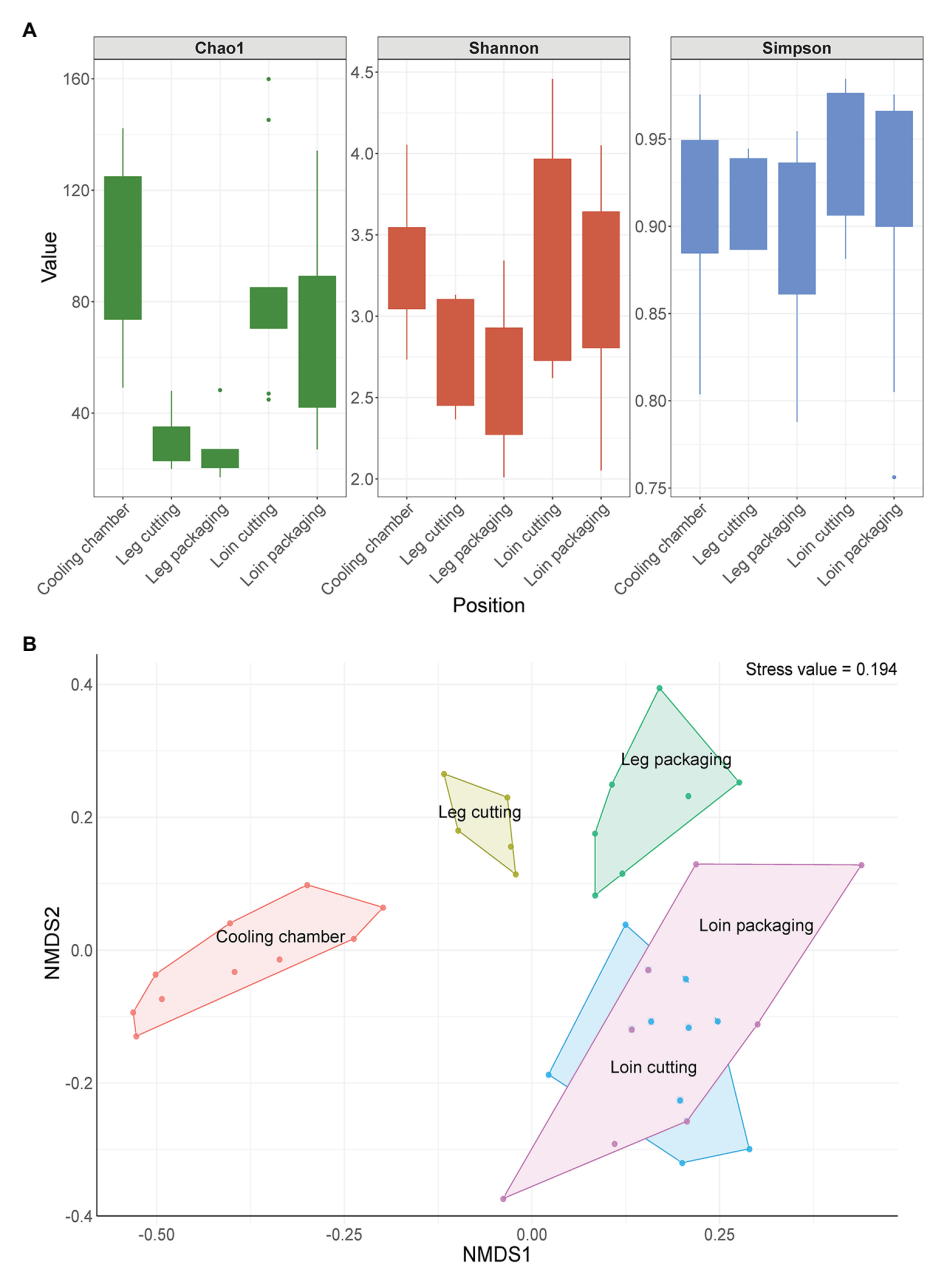
**(A)** Change in alpha diversity indices of meat samples over time. Boxes indicate the interquartile range (from 75 to 25th) of the data. Whiskers extend to the most extreme value within 1.5 times interquartile range and dots represent outliers beyond that range. **(B)** Non-metric multidimensional scaling (nMDS) plot of Bray-Curtis distances based on 16S ribosomal RNA (rRNA) gene libraries obtained from meat samples. Each point represents values from individual libraries with colors expressing meat samples from different positions along the processing line.

In total, 158,113 full-length 16S rRNA gene sequences and 1,373 ASVs passed our stringent quality, prevalence, and decontamination filtering thresholds, resulting in 1,777 sequences per sample on average. We estimated microbial biodiversity within samples using different alpha diversity indices (Chao1, Shannon, and Simpson index). Species richness (Chao1) was highest on meat samples taken at the beginning of the processing line and lowest for samples taken from the leg (Figure 3A). ASVs were similarly evenly distributed across all sampling positions, as shown by the Simpson index. The Shannon index shows that the species diversity was lower in leg samples compared to loin samples when abundance is considered. However, beta diversity analysis revealed that microbial communities on meat showed a strong patterning according to sampling position (Figure 3B). Samples taken at the cooling chamber cluster apart from leg and loin samples, which are also separated from each other. Interestingly, microbiomes of leg cutting samples were also different from microbiomes of leg packaging samples, whereas loin samples exhibited a similar microbial community structure throughout processing. To statistically test whether the microbial communities of different sample groups differ from each other, we calculated a permutational analysis of variance (Supplementary Table S1). The factors “Type” (meat or environmental sample) and “Position” (sampling positions) were the most significant factors (*p* < 0.001) responsible for differences in the microbiome. Moreover, the presence of listeria (Factor “Listeria”: present or not present) had a significant impact (*p* = 0.04879) on the structure of microbial communities. The factor “Carcass” (Meat from carcass A–C, etc.) was not significant, further substantiating that the microbiome of the same meat sample is not stable throughout processing and changes from start to end. The core microbiome on meat within the facility, defined here as all ASVs that occur in at least 10 samples with a minimum relative abundance of 0.01%, was comprised of 22 ASVs with a combined relative abundance of 17% across all samples (Supplementary Figure S3). Taxonomic classification of these 22 ASVs revealed *Acinetobacter*, *Pseudomonas*, *Psychrobacter* (*Proteobacteria*), *Bacillus* (*Firmicutes*), and *Chryseobacterium* (*Bacteroidota*) as the most represented genera (Supplementary Table S2).

### Taxonomic Composition of Meat and Surface Samples During Meat Processing

**Figure 4:**
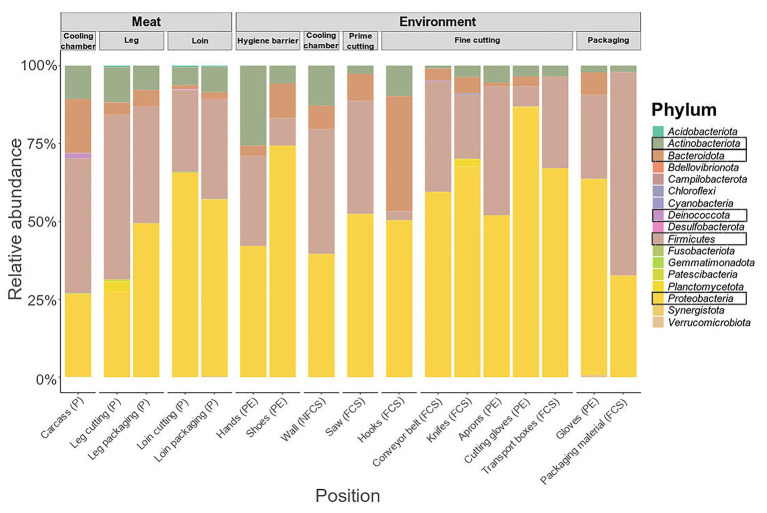
Phylum-level classification of 16S rRNA gene sequence reads parted by sampling position and type (meat or environment). Data represent average of amplicon sequence variant (ASV) counts from replicate libraries for each category. Sample categories are abbreviated: “product” (P), “food contact surface” (FCS), “non-FCS” (NFCS), and “personnel” (PE).

**Figure 5:**
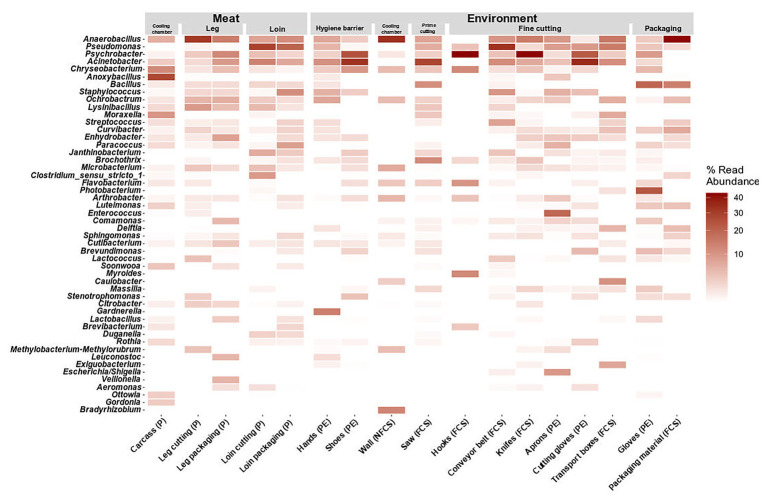
Heatmap of the top 50 most abundant genera grouped by position and split by type. Data represent average of ASV counts from replicate 16S rRNA gene libraries for each category. Sample categories are abbreviated: “product” (P), “food contact surface” (FCS), “non-FCS” (NFCS), and “personnel” (PE).

The microbiome on meat was dominated by *Firmicutes* (43.35%), *Proteobacteria* (26.50%), *Bacteroidota* (17.26%), and *Actinobacteriota* (10.76%) when the carcasses were delivered to the facility (cooling chamber, Figure 4). While *Actinobacteriota* (leg packaging: 7.76%; loin packaging: 8.22%) remained stable over the course of processing, the relative abundance of *Bacteroidota* (5.42%; 2.00%) and *Firmicutes* (37.42%; 32.01%) decreased and *Proteobacteria* (49.41%; 56.79%) increased toward the end of the processing line. Primary phyla on surface samples were *Firmicutes* and *Proteobacteria*, although they displayed a diverse microbial community in general (Supplementary Figure S4). To resolve these differences on a finer scale, we looked at the 50 most abundant genera across all samples (Figure 5).

At large, genera that were detected on the meat were also found in one or more additional environmental samples (*Pseudomonas*, *Psychrobacter*, *Acinetobacter*, *Chryseobacterium*, *Anaerobacillus*, and *Anoxybacillus*). *Pseudomonas* was not present initially in the cooling chamber (P and NFCS), but was strongly associated to loin cutting and packaging (P, FCS, and PE) and conveyor belts (FCS) indicating cross-contamination on these stations. *Psychrobacter* and *Acinetobacter* were highly present in the FPE (shoes, saw, hooks-leg, and knives) pointing out environmental reservoirs. *Anaerobacillus* was most often observed on walls of the cooling chamber, leg, and packaging material. *Bacillus* was detected at the saw and at the last station (packaging). Selective hotspots for specific microbiota members were observed on the carcass in the cooling chamber (*Anoxybacillus*), on hands at hygiene barrier (*Gardnerella*) on aprons at the fine cutting (*Enterococcus*), and gloves at packaging (*Arthrobacter*).

### The Presence of *Listeria* is Associated With Higher Abundances of Diverse Genera

**Figure 6:**
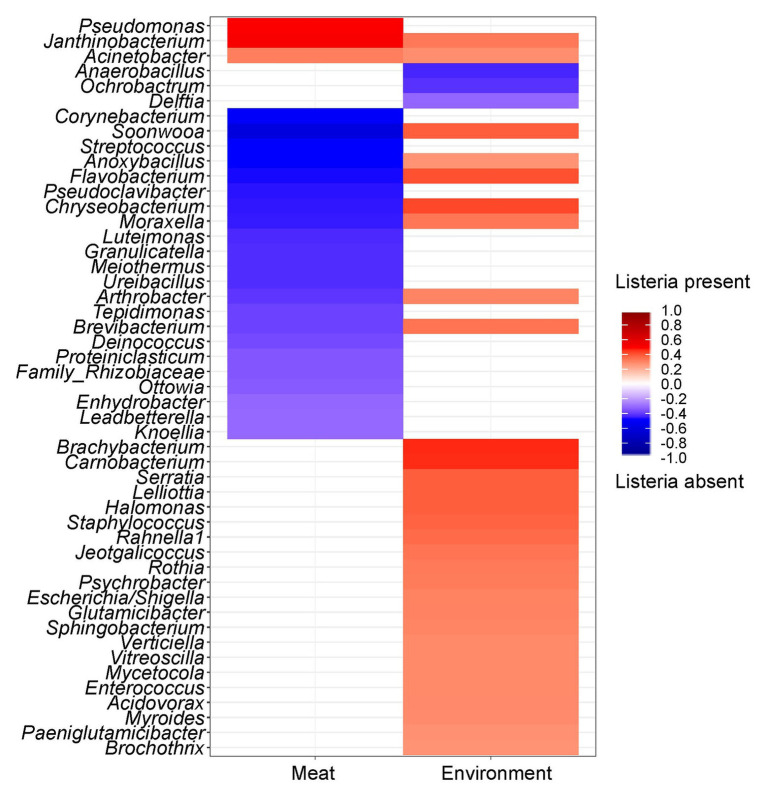
Heatmap showing the correlation of individual genera between samples with or without listeria. Red color shows a positive correlation and blue color illustrates a negative correlation with the presence of listeria. The saturation of the color indicates the strength of the correlation coefficient. Only significant (*p* ≤ 0.05) taxa are shown.

**Figure 7:**
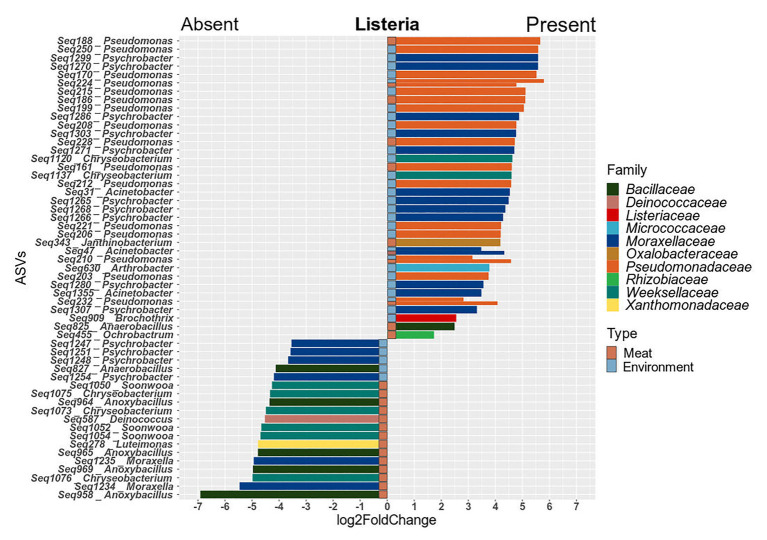
Barplots of ASVs that were significantly differentially abundant (*p* ≤ 0.05) between meat and surface samples with and without listeria. Positive values indicate higher abundance in samples with listeria and negative values depict higher abundance in samples where listeria is absent. Significant ASVs are plotted individually and colored according to their family-level classification.

Since the prevalence of listeria was identified as a significant factor explaining shifts in the microbial community structure, we were interested in which specific microorganisms were linked to the presence or absence of listeria. Thus, we calculated Spearman rank correlations for each individual taxon between the two sample groups (Figure 6). *Acinetobacter* and *Janthinobacterium* were positively associated with the presence of listeria on both meat and surface samples. Other bacteria, e.g., *Brachybacterium* and *Carnobacterium* were positively correlated to listeria only on surface samples. *Pseudomonas* was the only genus that showed positive correlation with listeria only in meat but not in environmental samples. *Chryseobacterium*, *Moraxella*, and *Anoxybacillus* (core microbiome) correlated positively with surface samples with listeria but negatively with meat samples with listeria. This opposing pattern might be explained by a varying behavior of different species or subpopulations of these genera. Thus, we looked into the differential abundance of individual ASVs, disregarding phylogenetic subsumptions. Our analysis revealed that *Chryseobacterium* ASVs (Seq1075, Seq1073, and Seq1076) were significantly higher abundant in meat samples in which listeria was absent, whereas other ASVs (Seq1120, Seq1137) were higher abundant in surface samples with listeria (Figure 7). Similarly, ASVs within the genus *Psychrobacter* had disparate associations with listeria in surface samples. Phylogenetic trees revealed that these ASVs represent diverse phylogenetic clades, suggesting that these interactions are species or even strain-specific (Supplementary Figures S5, S6).

Overall, ASVs that were significantly higher abundant in the processing environment in parallel with listeria were associated to the genera *Pseudomonas*, *Acinetobacter*, *Psychrobacter*, *Arthrobacter*, and *Brochothrix*. In meat samples, the genera *Pseudomonas*, *Acinetobacter*, *Janthinobacterium*, *Ochrobactrum*, and *Anaerobacillus* entailed ASVs that were co-occurring with listeria. Highly abundant *Pseudomonas* ASVs which could be assigned to species level were *P. fragi* (Seq203, 232, 210, 215, 195, 200, 222-meat, and environment), *P. psychrophila* (Seq228), and *P. fluorescens* (Seq161). The latter were abundant on hooks, conveyor belts, loin cutting, and packaging in parallel with *L. monocytogenes* PFGE profiles MA3-17, MA5-17, MA13-17, and MA14-17.

## Discussion

Frequent listeriosis outbreaks throughout the last decade notably underlined the necessity of research on the persistence and ecology of *L. monocytogenes* within diverse food processing plants (Buchanan et al., 2017). Many studies investigated *L. monocytogenes* prevalence and traced outbreaks using whole genome sequencing (WGS) of isolates (Jackson et al., 2016; Halbedel et al., 2018; Pietzka et al., 2019). However, more research that delivers community wide information is necessary in order to understand why *L. monocytogenes* was able to persist and contaminate food from FPE.

Currently, one of the main methods to inactivate *L. monocytogenes* in ready to-eat meat products is high pressure processing, although it can affect organoleptic properties of the meat (Teixeira et al., 2014). Developing effective risk management strategies is dependent on unraveling mechanisms of interactions between *L. monocytogenes* and the built-environment microbiome that contribute to the persistence and recontamination of FPE with *L. monocytogenes*. Here, we applied high-throughput full-length 16S rRNA gene sequencing to investigate the presence of *Listeria* spp. in a meat processing plant in the context of whole bacterial communities. Our results reveal co-occurrence of *Listeria* spp. and diverse members of the core community throughout processing.

The increase in AMC after the first processing steps indicate cross contamination events of microorganisms from the environment as well as the increase of psychrotrophic microbiota associated with the processed meat. Especially, at the leg cutting station AMC raised to critical limits (DGHM, 2014; >6.7 log CFU). The meat and environmental samples harbored higher *Pseudomonadaceae* in comparison to *Enterobacteriaceae* counts, except for the delivered carcass samples, after leg cutting and on shoes and on the wall of the cooling chamber (Supplementary Figure S2). However, species richness was lower at leg and loin cutting samples compared to samples taken at the beginning of the processing line (cooling chamber). In congruence with the shift in beta diversity, this indicates that the transfer of bacteria rapidly changes the overall composition of the microbiota between these steps to a community dominated by a few bacteria that are endemic in the facility. These results are in line with previous findings and together demonstrate that the facility-specific microbiome greatly affects the composition of the microbial community found on the final product (Bokulich and Mills, 2013; de Filippis et al., 2013; Hultman et al., 2015; Einson et al., 2018; Quijada et al., 2018; Zwirzitz et al., 2020). Among all samples, the primary phyla of surface samples were *Firmicutes* and *Proteobacteria*. In contrast, Rodríguez-López et al. (2020) identified *Actinobacteriota* and *Firmicutes* as the most dominant phyla in meat industry samples (Rodríguez-López et al., 2020).

The core in-house microbiota consisted mainly of the genera *Acinetobacter*, *Pseudomonas*, *Psychrobacter* (*Proteobacteria*), *Bacillus* (*Firmicutes*), and *Chryseobacterium* (*Bacteroidota*) which have been commonly isolated from other FPEs before (Stellato et al., 2015, 2016; Hascoët et al., 2019). Since all these genera are also highly abundant and include species that are frequently described as spoilage organisms, the widespread establishment of these bacteria throughout the facility is concerning (Odeyemi et al., 2020). In another study, bacterial communities from biofilms in four FPEs were characterized by 16S rRNA gene sequencing: *Proteobacteria*, *Firmicutes*, *Actinobacteriota*, and *Bacteroidota* represented over 94% of the operational taxonomic units, and *Pseudomonas* and *Acinetobacter* were the most dominant genera (93.47%; Caraballo Guzmán et al., 2020). *Pseudomonas* and *Acinetobacter* were the most frequently isolated genera surviving on conveyor belts after cleaning and disinfection in meat processing plants (Fagerlund et al., 2017). In our dataset, *Pseudomonas* was besides *Anaerobacillus* the most abundant genus throughout meat cutting and was involved in cross-contamination events from the environment. In detail, *P. fragi, P. psychrophila*, and *P. fluorescens* could be assigned to species level and were strongly linked to the FPE and meat cuts. This is in line with the findings from Stellato et al. (2017), who determined the most dominant *Pseudomonas* species in both meat and dairy processing environments (Stellato et al., 2017).

Listeria testing of meat, environmental, and personnel associated samples revealed a widely disseminated *L. monocytogenes* contamination (50% of samples *Listeria* spp. positive, 6% *L. monocytogenes* positive) throughout processing. Similar prevalence levels of listeria were observed in other processing environments as well (Muhterem-Uyar et al., 2015). Listeria was absent from carcass samples, hooks and wall in the cooling chamber, but was present due to cross-contamination events during meat cutting and packaging. The most prevalent *L. monocytogenes* serotype was 1/2a, 3a and was highly present in the FPE (Stessl et al., 2020). The PFGE typing evidenced 14 heterogenous *L. monocytogenes* profiles, with PFGE profile MA3-17 as most dominant. In future studies, listeria loads should be repeatedly recorded in order to get quantitative data and insights into the stability of the listeria population. To mitigate listeria contamination, operators could determine listeria levels prior to the supply to meat processing plants, using pooled surface samples per carcass and production environment. Carcasses from suppliers that more frequently contain low levels of listeria should not enter the processing area of low-processed edible meat products. In processing plants, where both heated and non-heated meat products are processed, environmental sampling for listeria should be intensified, with a focus on crossing points.

Pulsed-field gel electrophoresis MA3-17 contributed to the resilient microbiota of the facility environment and was related to environmental persistence as shoes after passing the hygiene lock were already positively tested. The hooks of the leg indicated inefficient sanitation due to the presence of up to four different PFGE-profiles and no *L. monocytogenes* negative sample. Saw and conveyor belts contained heterogenous *L. monocytogenes* PFGE-types. Furthermore, packaging material was tested positive for listeria highlighting the need of treating the packaging materials as a potential source of cross-contamination (Di Ciccio et al., 2020). Other studies compared the microbiome and the presence of *L. monocytogenes* in different FPEs and highlighted meat facilities as a common source of *L. monocytogenes* (Rodríguez-López et al., 2019). A wide variety of phylogenetic taxa co-occurred with *L. monocytogenes* (*Psychromonas*, *Shewanella*, *Yersinia*, and lactic acid bacteria). The authors concluded that *L. monocytogenes* is capable to co-exist with different bacteria in different ecological niches. Tan et al. (2019) observed *L. monocytogenes* persistence in the facility environment of an apple processing plant which was correlating with reduced bacterial diversity (*Pseudomonas* predominant) in comparison to other plants (Tan et al., 2019).

In general, the presence of listeria had a significant impact on the microbial community structure, affecting multiple taxa across the phylogenetic tree. Future studies are necessary to elucidate whether the presence of listeria drives these shifts in the microbial community, or rather the composition of the pre-existing community fosters suitable conditions for listeria to thrive. However, the ability of *L. monocytogenes* to survive and persist in biofilms of other bacteria has been proposed by others before (Fagerlund et al., 2017; Heir et al., 2018). Furthermore, Puga et al. (2018) have shown that *L. monocytogenes* is able to colonize biofilms formed by *P. fluorescens* in co-culture experiments leading to higher bacterial cell densities *in vitro* (Puga et al., 2018). Our study confirms that the same colonization progression occurs in the environment. We propose to perform similar experiments with the other bacteria that had positive correlations with listeria in this study. Especially, *Acinetobacter* and *Janthinobacterium*, another bacterium that had significantly higher abundances when listeria was present, are known to form biofilms and would be prime prospects to test more complex ecological interactions in controlled experiments. Based on our data, we hypothesize that listeria persists in multispecies biofilms formed by a consortium of *Pseudomonas*, *Acinetobacter*, *Janthinobacterium*, and other species in FPE.

## Conclusion

Understanding how individual microbial community members of various FPE interact with each other will give us deeper insights into improved and targeted disinfection strategies. Diverse correlations from ASVs belonging to the same genus support that these interactions are highly specific and that a high taxonomic resolution is necessary when performing metagenomic analysis. Overall, our study suggests that interactions and symbiosis of microorganisms in addition to inherent genetic and environmental factors contribute to listeria persistence in FPE. Metagenomic and metatranscriptomic analysis as well as *in vitro* experiments are needed to gain more knowledge about the exact metabolic interactions and co-dependence of certain species and strains during biofilm formation and development in regard to *L. monocytogenes* prevalence.

## Data Availability Statement

The datasets presented in this study can be found in online repositories. The names of the repository/repositories and accession number(s) can be found at: https://www.ebi.ac.uk/ena, PRJEB38260.

## Author Contributions

BZ, SW, MW, and ES conceived and designed the study. Sampling was performed by BZ, SW, and BS. BZ, ST, MD, and BS performed the experiments. BZ, ED, and NQ developed bioinformatics pipelines. Data analysis and statistics were performed by BZ. BZ and BS wrote the manuscript. All authors contributed to the article and approved the submitted version.

### Conflict of Interest

The authors declare that the research was conducted in the absence of any commercial or financial relationships that could be construed as a potential conflict of interest.

## Footnotes

^1^https://www.pacb.com/wp-content/uploads/Procedure-Checklist-Full-Length-16S-Amplification-SMRTbell-Library-Preparation-and-Sequencing.pdf

^2^www.vbcf.ac.at

^3^https://github.com/PacificBiosciences/pbbioconda
