# Supplementary material for: Co-Occurrence of Listeria spp. and Spoilage Associated Microbiota During Meat Processing Due to Cross-Contamination Events
Source: Front Microbiol. 2021 Feb 5;12:632935. doi: 10.3389/fmicb.2021.632935 (PMC7892895; doi:10.3389/fmicb.2021.632935)
Supplement: Supplementary file 1 [file Data_Sheet_1.docx]

Supplementary Material


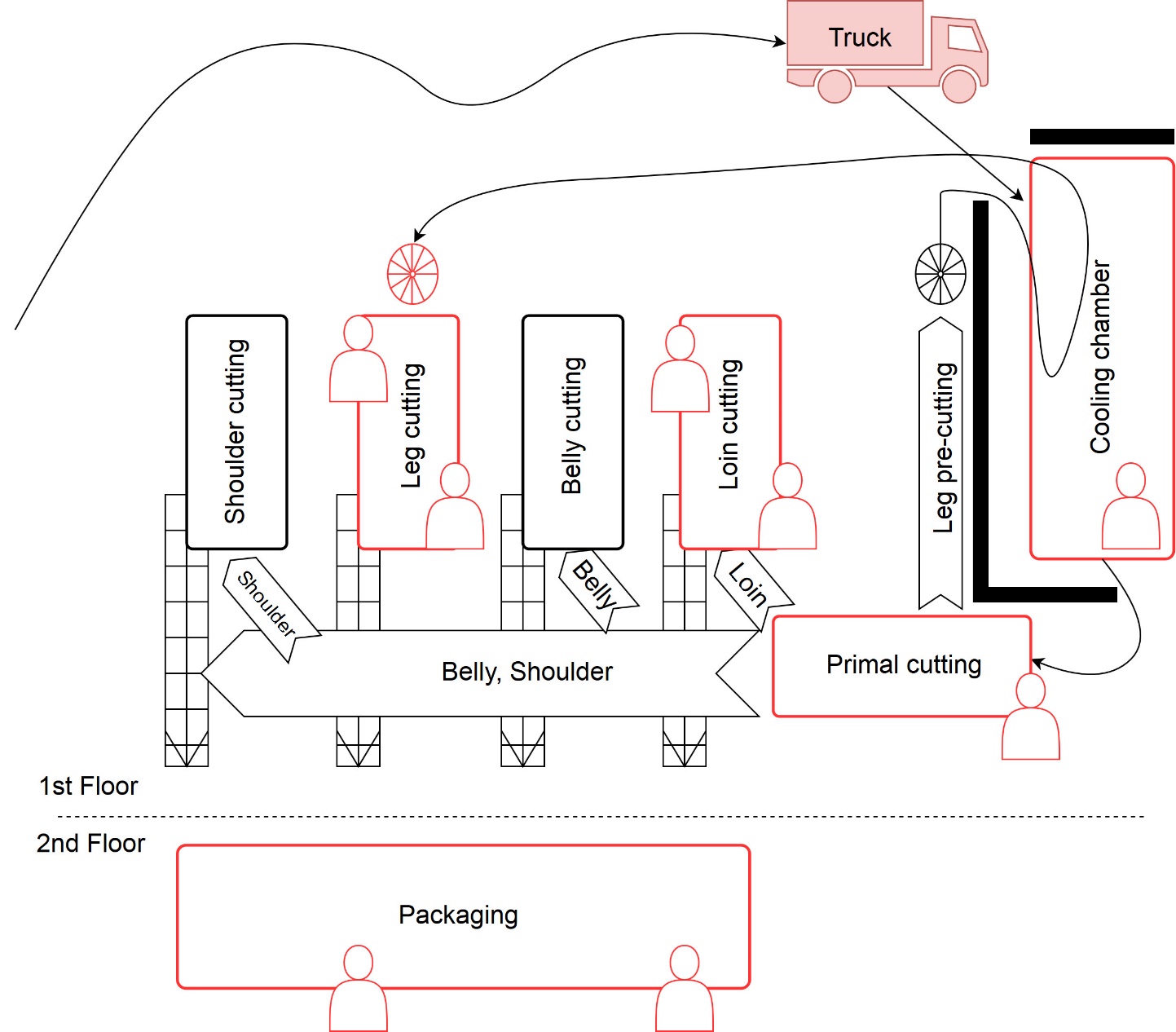
Supplementary Figure S1. Schematic map of the facility. Sampling positions are indicated in red colour.

**
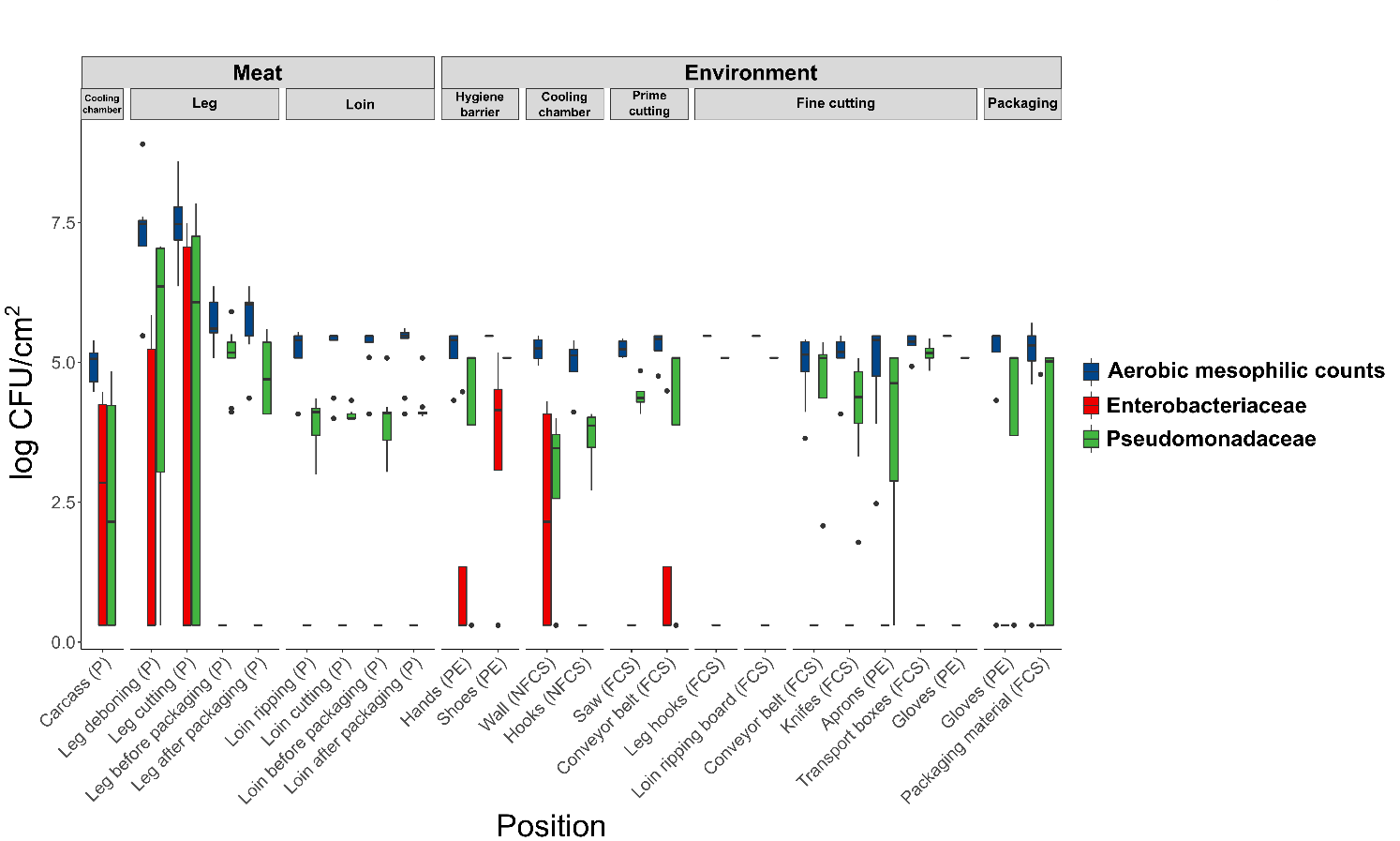
**

**Supplementary Figure S2.** Quantification of aerobic mesophilic counts (AMC), Enterobacteriaceae (EB), and Pseudomonadaceae. Boxes indicate the interquartile range (75th to 25th) of the data. The median is shown as a line within the box. Whiskers extend to the most extreme value within 1.5 * interquartile range. Sample categories are abbreviated: “Product” (P), “Food Contact Surface” (FCS), “Non-Food Contact Surface” (NFCS), and “Personnel” (PE).

| **Test group** | **Df** | **SumsOfSqs** | **MeanSqs** | **F.Model** | **R2** | **Pr(>F)** |
| --- | --- | --- | --- | --- | --- | --- |
| **Type** | 1 | 1.037 | 1.03672 | 2.63964 | 0.0576 | <0.001 *** |
| **Position** | 15 | 10.944 | 0.72962 | 1.85770 | 0.27192 | <0.001 *** |
| **Listeria** | 1 | 0.495 | 0.49523 | 1.26093 | 0.01230 | 0.04879 * |
| **Carcass** | 9 | 3.422 | 0.38021 | 0.96805 | 0.08502 | 0.74505 |
| **Residuals** | 62 | 24.351 | 0.39275 |  | 0.60500 |  |
| **Total** | 88 | 40.249 |  |  | 1 |  |

**Supplementary Table S1.** Results from a PERMANOVA test for the inﬂuence of sampling groups. Df = Degrees of Freedom. Significance levels: p≤0.001 = ***, p≤0.01 = **, p≤0.05 = *.


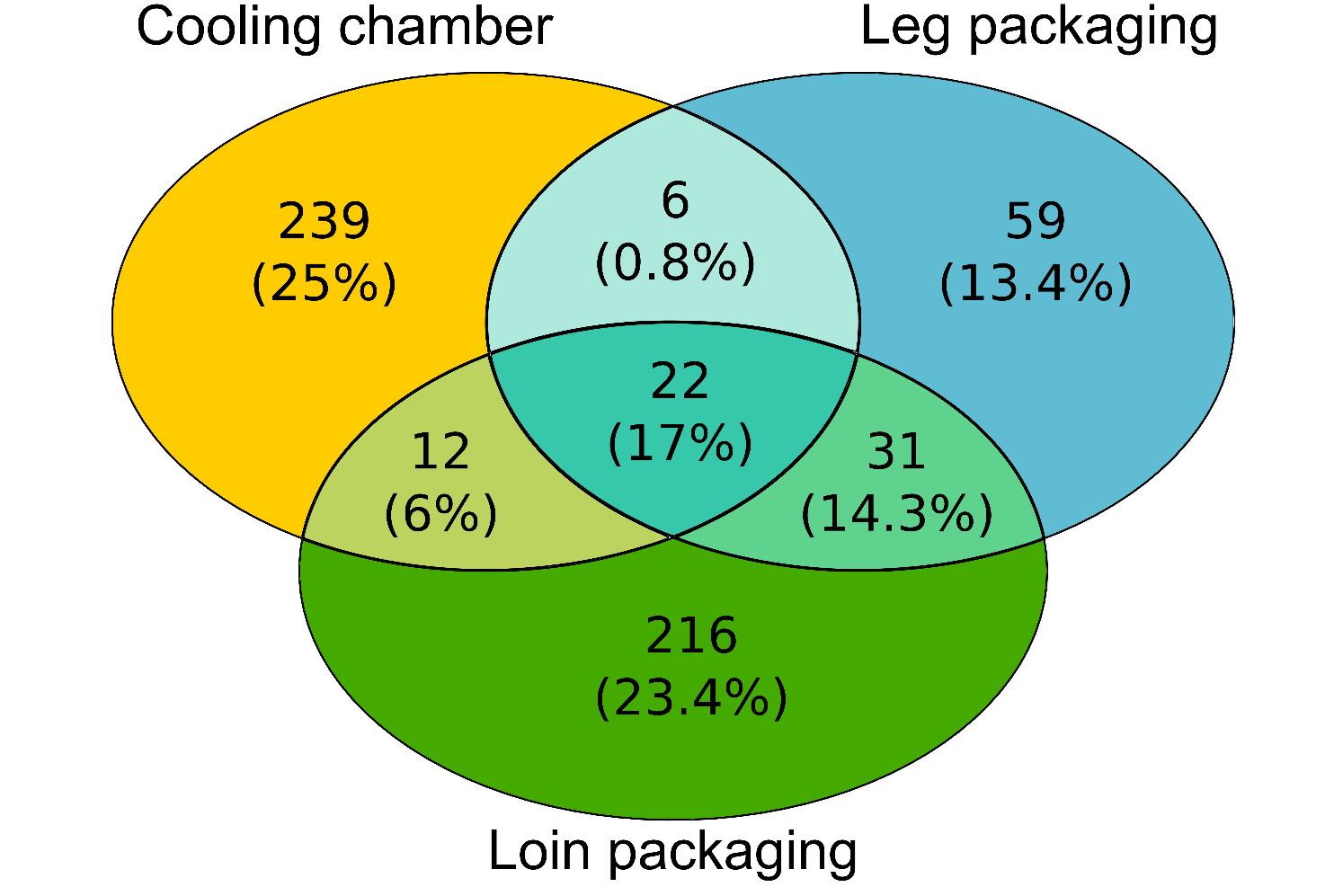


**Supplementary Figure S3.** Venn diagram of shared ASVs between meat samples from the start and the end of the processing line. Numbers depict the number of shared ASVs and percentages show the combined relative abundance of the shared ASVs.

| **ASV** | **Phylum** | **Class** | **Order** | **Family** | **Genus** | **Species** |
| --- | --- | --- | --- | --- | --- | --- |
| **Seq385** | Proteobacteria | Gammaproteobacteria | Pseudomonadales | Moraxellaceae | Acinetobacter | Genus_Acinetobacter |
| **Seq455** | Proteobacteria | Gammaproteobacteria | Pseudomonadales | Moraxellaceae | Acinetobacter | Genus_Acinetobacter |
| **Seq495** | Proteobacteria | Gammaproteobacteria | Pseudomonadales | Moraxellaceae | Enhydrobacter | Genus_Enhydrobacter |
| **Seq679** | Bacteroidota | Bacteroidia | Flavobacteriales | Weeksellaceae | Chryseobacterium | Genus_Chryseobacterium |
| **Seq700** | Actinobacteriota | Actinobacteria | Micrococcales | Microbacteriaceae | Leucobacter | Genus_Leucobacter |
| **Seq797** | Proteobacteria | Alphaproteobacteria | Rhodobacterales | Rhodobacteraceae | Paracoccus | Genus_Paracoccus |
| **Seq810** | Proteobacteria | Gammaproteobacteria | Pseudomonadales | Moraxellaceae | Acinetobacter | Genus_Acinetobacter |
| **Seq812** | Actinobacteriota | Coriobacteriia | Coriobacteriales | Atopobiaceae | Atopobium | Genus_Atopobium |
| **Seq814** | Proteobacteria | Gammaproteobacteria | Pseudomonadales | Moraxellaceae | Moraxella | Genus_Moraxella |
| **Seq820** | Proteobacteria | Gammaproteobacteria | Pseudomonadales | Moraxellaceae | Psychrobacter | Genus_Psychrobacter |
| **Seq822** | Firmicutes | Clostridia | Peptostreptococcales-Tissierellales | Peptostreptococcales-Tissierellales_fa | Peptoniphilus | Genus_Peptoniphilus |
| **Seq823** | Firmicutes | Bacilli | Lactobacillales | Aerococcaceae | Aerococcus | Genus_Aerococcus |
| **Seq828** | Proteobacteria | Gammaproteobacteria | Pseudomonadales | Pseudomonadaceae | Pseudomonas | Genus_Pseudomonas |
| **Seq835** | Proteobacteria | Gammaproteobacteria | Xanthomonadales | Xanthomonadaceae | Stenotrophomonas | Genus_Stenotrophomonas |
| **Seq848** | Proteobacteria | Alphaproteobacteria | Sphingomonadales | Sphingomonadaceae | Sphingomonas | Genus_Sphingomonas |
| **Seq910** | Bacteroidota | Bacteroidia | Flavobacteriales | Weeksellaceae | Chryseobacterium | Genus_Chryseobacterium |
| **Seq977** | Bacteroidota | Bacteroidia | Flavobacteriales | Weeksellaceae | Chryseobacterium | Genus_Chryseobacterium |
| **Seq980** | Firmicutes | Clostridia | Peptostreptococcales-Tissierellales | Peptostreptococcaceae | Romboutsia | Genus_Romboutsia |
| **Seq981** | Firmicutes | Bacilli | Lactobacillales | Streptococcaceae | Streptococcus | dysgalactiae |
| **Seq1209** | Firmicutes | Bacilli | Bacillales | Bacillaceae | Bacillus | Genus_Bacillus |
| **Seq1259** | Actinobacteriota | Actinobacteria | Micrococcales | Intrasporangiaceae | Janibacter | Genus_Janibacter |
| **Seq1262** | Proteobacteria | Alphaproteobacteria | Rhodobacterales | Rhodobacteraceae | Family_Rhodobacteraceae | Family_Rhodobacteraceae |

**Supplementary Table S2.** List of ASVs (n=22) found to be part of the core community with their associated taxonomy.


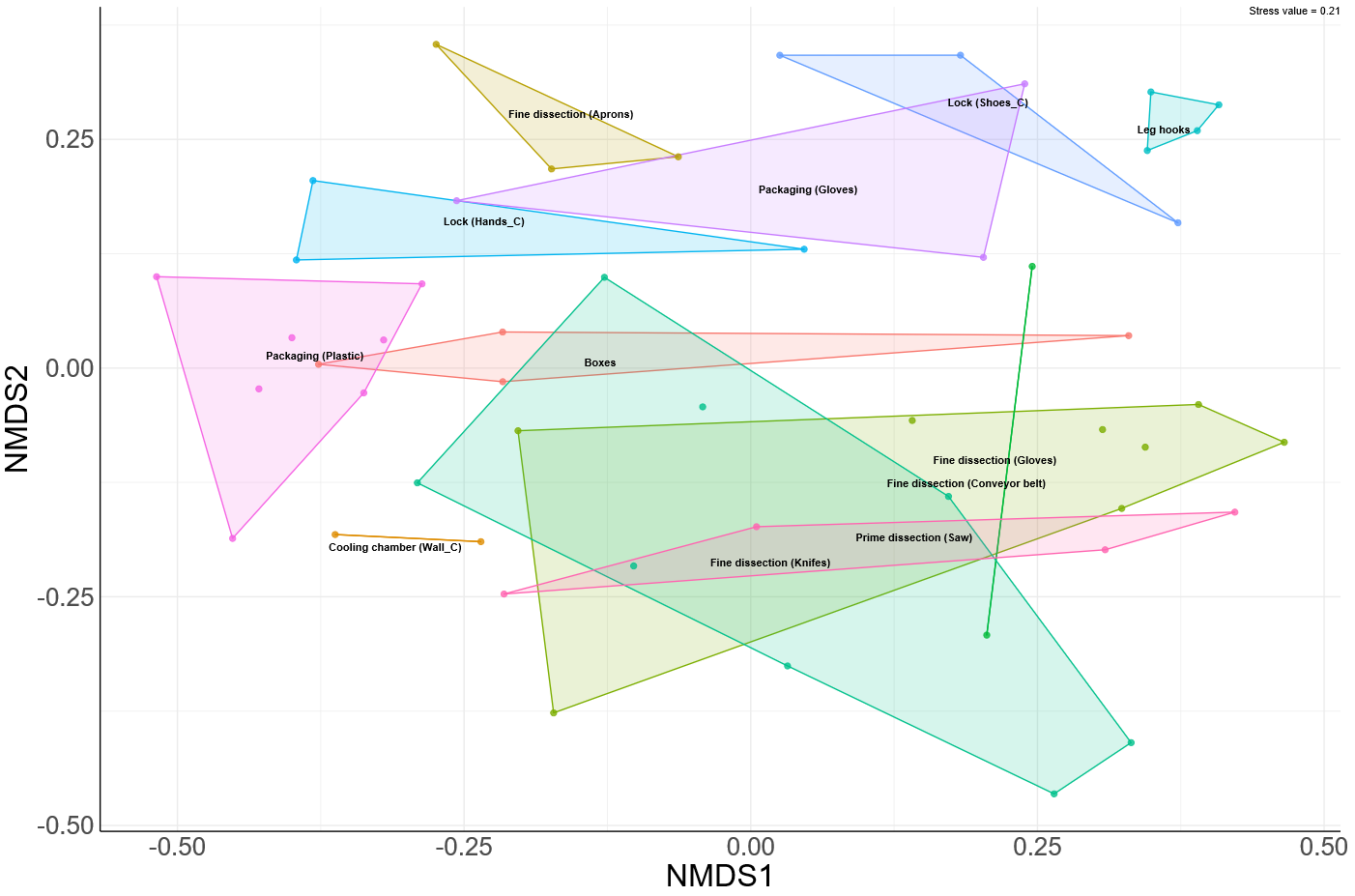
**Supplementary Figure S4.** Non-metric multidimensional scaling (nMDS) plot of Bray-Curtis distances based on 16S rRNA gene libraries obtained from surface samples. Each point represents values from individual libraries with colors expressing surface samples from different positions along the processing line.


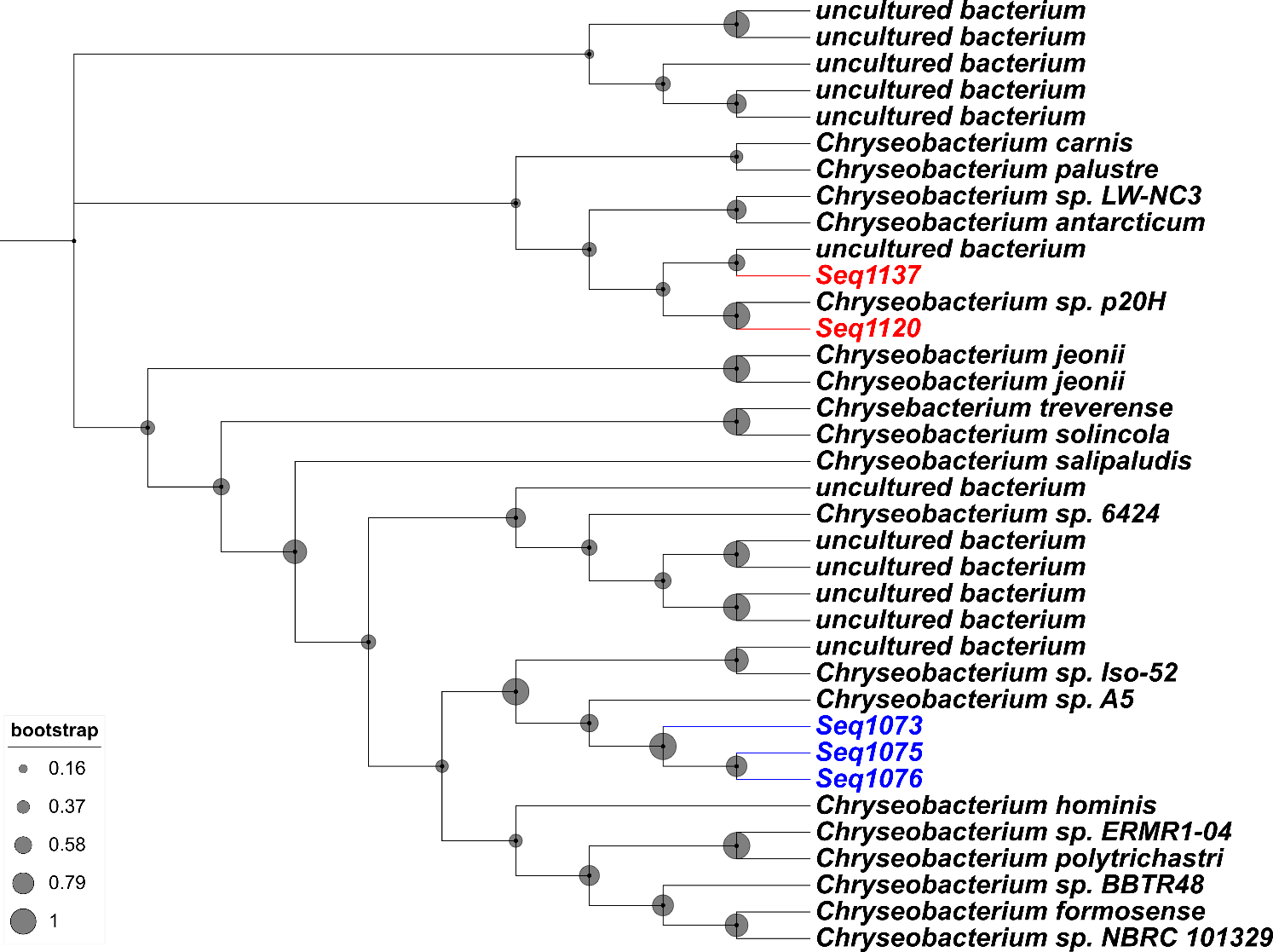
**Supplementary Figure S5.** Maximum likelihood tree of 16S rRNA gene sequences of all ASVs associated to the genus Chryseobacterium that were identified to be significantly different between samples with and without listeria. Their 25 closest neighbors were included for tree generation. ASVs in red are higher abundant when listeria is present and ASVs labelled in blue are higher abundant when listeria was absent. Bootstrap values are based on 500 bootstrap replicates.


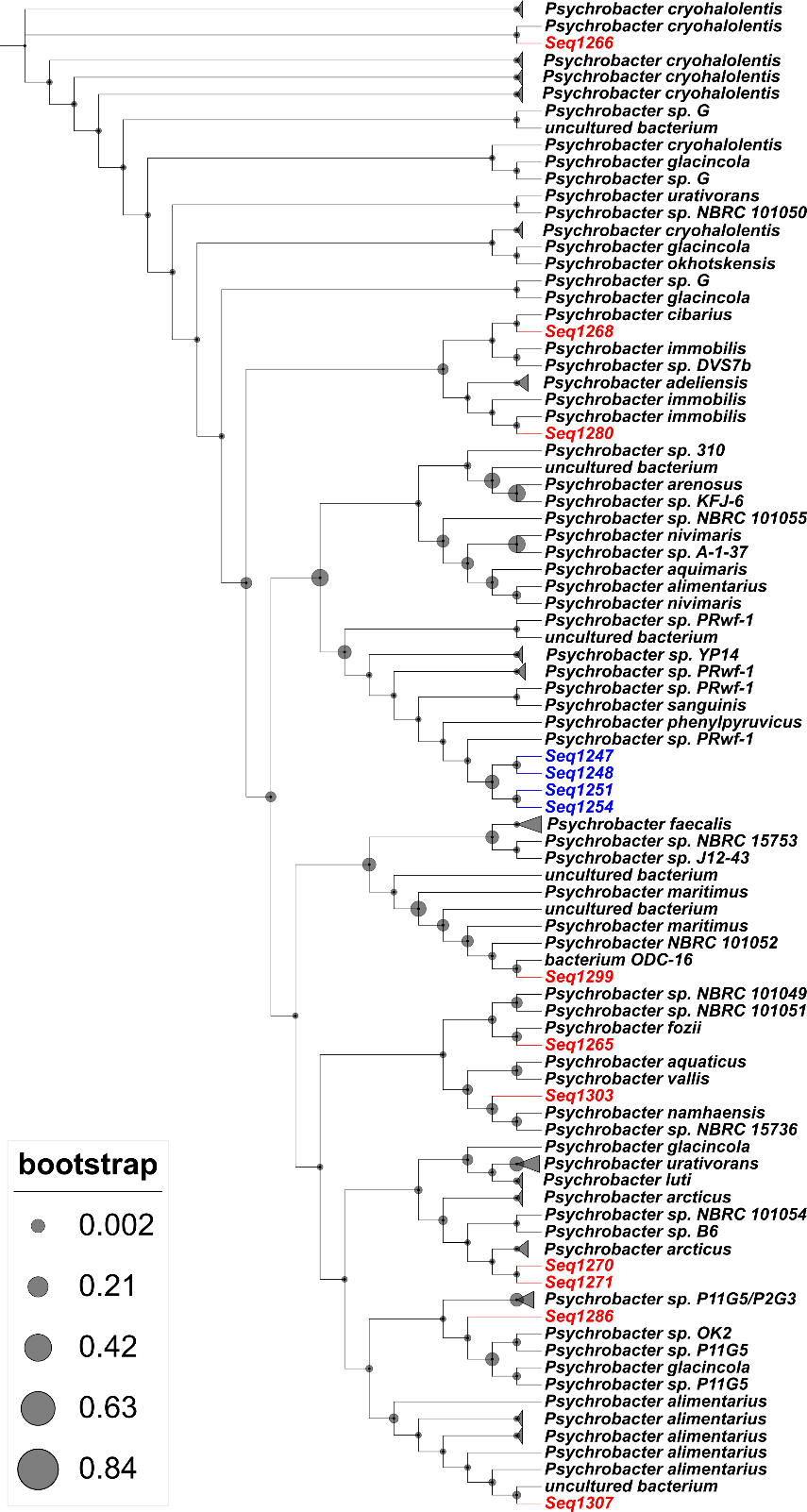


**Supplementary Figure S6.** Maximum likelihood tree of 16S rRNA gene sequences of all ASVs associated to the genus Psychrobacter that were identified to be significantly different between samples with and without listeria. Their 25 closest neighbors were included for tree generation. ASVs in red are higher abundant when listeria is present and ASVs labelled in blue are higher abundant when listeria was absent. Bootstrap values are based on 500 bootstrap replicates.
